# Serum vitamin D levels are associated with structural and functional properties of the carotid artery in older men and women

**DOI:** 10.1007/s41999-020-00296-0

**Published:** 2020-02-21

**Authors:** Christian Oudshoorn, Martina Mezzadri, Edgar M. Colin, Suzanne C. van Dijk, Astrid G. Ruitenbeek, Anton H. van den Meiracker, Tischa J. M. van der Cammen, Francesco U. S. Mattace-Raso

**Affiliations:** 1 Division of Geriatric Medicine, Department of Internal Medicine, Erasmus MC University Medical Center, Rotterdam, The Netherlands; 2 Department of Rheumatology, Erasmus MC University Medical Center, Rotterdam, The Netherlands; 3 Division of Pharmacology and Vascular Medicine, Department of Internal Medicine, Erasmus MC University Medical Center, Rotterdam, The Netherlands

**Keywords:** Serum vitamin D levels, Carotid artery distensibility, Brachial artery distensibility, Atherosclerosis, Arterial stiffness, Cardiovascular disease

## Abstract

**Background and aims:**

This cross-sectional study aimed to assess the relationship between serum vitamin D levels and carotid and brachial artery distensibility in patients older than 55 years, referred to the outpatient clinic of the department of internal medicine and geriatric medicine of the Erasmus Medical Center, in Rotterdam.

**Methods and results:**

From April to July 2006 we consecutively enrolled 49 elder patients (21 men and 28 women, mean age 78 ± 8 years) without a cardiovascular event within 6 weeks before the visit. Carotid and brachial artery distensibility coefficients and serum 25(OH)D levels (mean 50 ± 28.8 nmol/L) were assessed. Multivariate analysis (with linear regression model) was performed to investigate the relation between these parameters: carotid artery distensibility coefficient was associated with serum 25(OH)D levels (*β* = 0.112; 95% CI 0.053 0.172; *p* = 0.001). Moreover, a negative association was also observed between carotid artery distensibility coefficient and mean arterial pressure (*β* = −0.279; 95% CI, −0.339 −0.159; *p* = 0.0001). On the contrary, brachial artery distensibility has shown no association with 25(OH)D levels, being negatively linked to LDL-cholesterol levels and heart rate. An association was also observed between serum 25(OH)D level and carotid artery intima–media thickness.

**Conclusion:**

Our results revealed that serum 25(OH)D levels of older men and women were associated with both structural and functional properties of the carotid artery. No association was found with the brachial artery distensibility.

## Key summary points

**Aim:**

To investigate the possible relation between serum vitamin D levels and arterial structural and functional properties in older men and women.

**Findings:**

Serum vitamin D levels are associated with carotid IMT and carotid stiffness. No assciation was found with brachial artery measurements.

**Message:**

The associations between serum vitamin D levels and arterial parameters seems to be driven by the type of artery; only elastic arteries show this association.

## Introduction

Vitamin D, a seco-steroid hormone, is mostly known to control skeletal patho/physiology, regulating calcium and phosphorus and bone remodelling. It is obtained through cutaneous synthesis resulting from sun exposure and through oral intake. It is metabolized first to 25-hydroxyvitamin D (25OH-D), then to the hormonal form 1,25-dihydroxyvitamin D (1,25(OH)_2_D). Analogs of 1,25(OH)_2_D are being developed to target specific diseases with minimal side effects [1].

25-hydroxyvitamin D (25-OH D) is considered to be the best indicator of vitamin D status in those with normal kidney function and reflects the level of circulating substrate. According to international literature, normal values of vitamin D is in a range from 30 to 40 ng/mL (75–100 nmol/L).

More recently, vitamin D deficiency has been considered as a risk factor for cardiovascular disease (CVD) and overall mortality in the general population.

Although the relationship between the traditional role of vitamin D in bone and calcium metabolism has been extensively studied, the mechanisms by which vitamin D deficiency confers vascular risk remain uncertain.

Recent publications, but only a small number of studies, suggest that vitamin D deficiency is also associated with and increased risk of aortic stiffness [2]. Previously, we found that central and peripheral arteries can be affected differently in regards to structural and functional properties [3]. In addition, if vascular stiffening (i.e., a decrease in vascular distensibility) is associated with vitamin D status and if there is a difference between various vascular regions is still unclear.

The aim of the present study is to evaluate whether mean serum vitamin D levels are associated with structural and functional arterial properties in a cohort of older persons.

## Individuals and methods

### Study design

From April to July 2006 we consecutively enrolled 49 patients (21 men and 28 women, mean age 78 ± 8 years), referred to the outpatient clinic of the Department of Internal Medicine and Geriatric Medicine, Erasmus MC University Medical Center, Rotterdam.

Patients aged 55 years and older were included and those with any cardiovascular event within 6 weeks before the visit were excluded from the study.

All individuals gave their informed consent before the study began. Information on previous cardiovascular disease, smoking habits, and drug use was obtained by interview from overall individuals. Patients were classified as ever-smokers (current or past smokers) or never-smokers.

This study was conducted in accordance with the Declaration of Helsinki guidelines: study design was clearly written in a layperson language and provided to each study participant. Written informed consent was obtained from all patients.

### Anthropometric measurements

Anthropometrics were measured in all individuals. Patients’ height and weight were collected, and body mass index [BMI; weight (kg)/height^2^ (m)] was calculated.

Standing height was measured in bare feet to the nearest 0.5 cm. Weight was measured in light clothing with a platform scale to the nearest 200 g. The scale was standardized to 0 before each use.

### Biochemical measurements

Diabetes mellitus was defined as a fasting serum glucose level equal to or greater than 7.0 mmol/L or as the use of blood glucose-lowering medication. Individuals were comfortably lying in a clinostatic position for at least 5 min and all venous samples were done in the morning (between 8:00 and 9:00 a.m.).

Serum 25(OH)D levels were measured using a radio-immunoassay (DiaSorin) and we considered as normal values of vitamin D a range from 30 to 40 ng/mL (75–100 nmol/L). Blood samples were collected the same day as the cardiovascular measurements were performed.

### Blood pressure measurements

Blood pressure and heart rate were measured twice on the right arm using an automatic device (Accutorr Plus; Datascope Corporation, Mahwah, New Jersey). Mean arterial pressure (MAP) was collected and the pulse pressure (PP) was calculated as systolic blood pressure (SBP)–diastolic blood pressure (DBP). The average of the 2 measurements was used in the analysis.

### Arterial distensibility measurements

After at least 5 min of rest with the patient in the supine position, arterial distensibility measurements were performed by Wall Track System 2 (Pie Medical, Maastricht, the Netherlands), using a B-mode ultrasound to identify the right common carotid artery at 1–2 cm proximal to the origin of the bulb. The right brachial artery was investigated at the antecubital crease. The end-diastolic diameter (*D*), the absolute stroke change in diameter during systole (Δ*D*), and the relative stroke change in diameter (Δ*D*/*D)* were computed as the mean of values measured in 4 s of 3 successive recordings. The distensibility coefficient (DC) was calculated by the following equation: 2(Δ*D*/*D*)/PP (10 MPa–1). The means of diameter and distension of 3 successive recordings were taken as the subject’s readings [4].

### Carotid artery intima–media thickness measurement

Carotid intima–media thickness For carotid B-mode ultrasonography, the L105 40 mm 7.5 MHz array transducer was used (Picus, Pie Medical Equipment, Maastricht, the Netherlands) on the right carotid artery. IMT is evaluated as the distance luminal–intimal interference and the media–adventitial interface (Art.Lab, Esoate Europe, Maastricht, the Netherlands) at approximately 1 cm from the carotid bifurcation.

### Statistical analysis

All data are expressed as mean ± standard deviation (± SD) and as percentages (%) for continuous and categorical variables, respectively. Multiple linear regression analysis was made with Backward Stepwise Regression method. A multivariate regression model was used to investigate the possible associations between serum 25(OH)D levels and the examined cardiovascular parameters (dependent variables). Models were adjusted for age, gender, MAP and heart rate. *p* values less than 0.05 were taken as statistically significant. Statistical analysis was performed using dedicated statistical software SPSS (Statistical Package for Social Sciences, software, version 2.; SPSS Inc, Chicago, Illinois, USA).

## Results

**Fig. 1:**
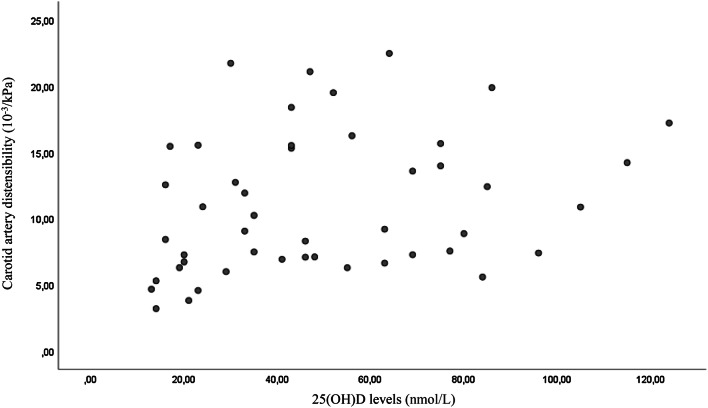
Scatter plot of the association between carotid artery distensibility and 25(OH)D levels (*p* = 0.001)

**Fig. 2:**
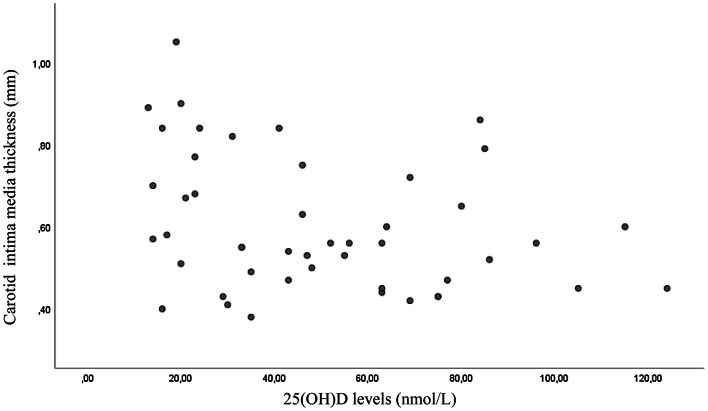
Scatter plot of the association between carotid intima–media thickness and 25(OH)D levels (*p* = 0.038)

**Fig. 3:**
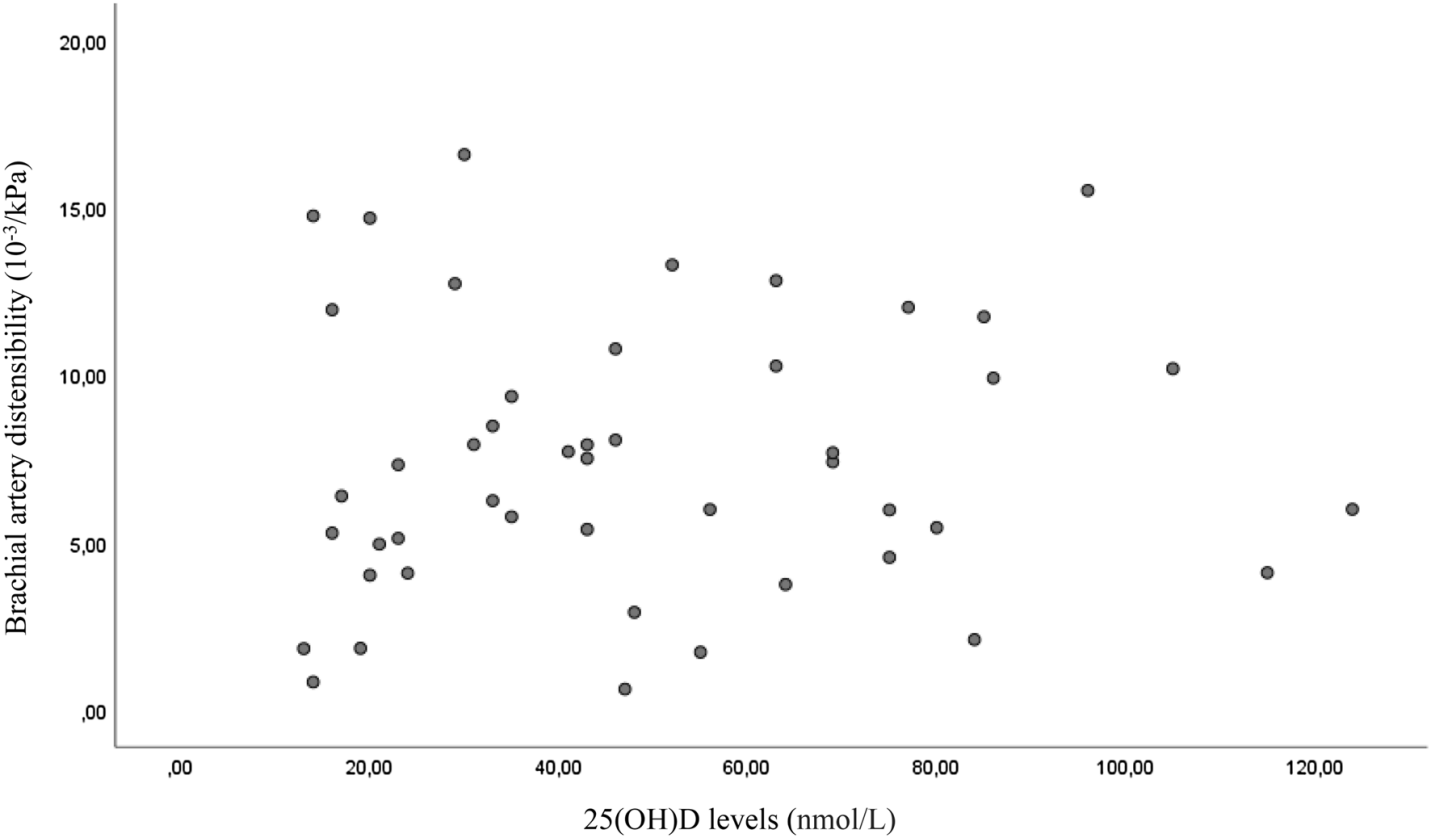
Scatter plot that shows no association between brachial artery distensibility and 25(OH)D levels (*p* = 0.51)

A total of 49 individuals, 21 men and 28 women, were evaluated in the present study, after the exclusion of patients younger than 55 years and those with any cardiovascular event within 6 weeks before the visit. The anthropometric, biochemical and instrumental characteristics of the study population are reported in Table 1. The mean systolic blood pressure was 131 ± 15.5 mmHg, and the mean diastolic blood pressure was 71 ± 9.1 mmHg. The mean serum 25(OH)D level was 50 ± 28.8 nmol/L. In this present study cohort, 28 of 49 participants (57%) had serum 25(OH)D levels ≤ 50 nmol/L and 39 out of 49 (80%) had serum 25(OH)D levels ≤ 75 nmol/L. Serum 25(OH)D levels were associated with carotid distensibility and carotid IMT but not with brachial distensibility (Figs. 1, 2, 3). In multivariate models an association was found between serum 25(OH)D levels and carotid artery distensibility (*β* = 0.048; 95% CI 0.010 0.096; *p* = 0.04), serum 25(OH)D levels) and carotid artery IMT (*β* = −0.002; 95% CI − 0.003 0.000; *p* = 0.048). No association was found between serum 25(OH)D levels and brachial artery distensibility (*β* = 0.013; 95% CI − 0.030 0.056; *p* = 0.135) (Table 2). Age was not associated with carotid distensibility, brachial distensibility or carotid intima–media thickness (data not shown.

**Table 1:** Baseline characteristics and biochemical laboratory values in overall patients enrolled (mean ± standard deviation)

Characteristic	Mean ± SD (*N *= 49)	Minimum	Maximum
Age (years)	77.93 ± 7.81	55.31	91.84
BMI (kg/m^2^)	25.6 ± 5.4	17.30	43.50
Waist circumference (cm)	38.09 ± 13.43	74	137
Smoking pack (/years)	16.92 ± 22	0	96.3
Exercise (hours/week)	1.75 ± 3.5	0	14
Intake of alcohol (units/week)	3.32 ± 5.53	0	30
SBP (mmHg)	130.8 ± 15.5	104.5	179.5
DBP (mmHg)	71.1 ± 9.1	50.00	93.5
HR (beats/min)	66.2 ± 11.2	37.00	89.00
PP (mmHg)	59.7 ± 11.4	42.5	96.5
MAP (mmHg)	94 ± 11.8	76.00	126.5
IMT (mm)	0.6 ± 0.16	0.38	1.05
Carotid distensibility (10^−3^/kPa) (coefficient)	11.0 ± 5.23	3.19	22.47
Brachial distensibility (10^−3^/kPa) (coefficient)	7.48 ± 4.1	0.62	16.69
Serum 25(OH)D3 levels (nmol/L)	50.3 ± 28.8	13.00	124.00
Total cholesterol (mmol/L)	5.56 ± 1.03	4	7.3
LDL-cholesterol (mmol/L)	3.36 ± 1.02	1.5	5.33
HDL-cholesterol (mmol/L)	1.96 ± 0.85	0.78	5.00
Triglycerides	1.55 ± 0.75	0.42	3.3
Glucose (mmol/L)	5.07 ± 1.76	2.4	13.1

*BMI* body mass index, *SBP* systolic blood pressure, *DBP* diastolic blood pressure, *HR* heart rate, *PP* pulse pressure, *MAP* mean arterial pressure, *IMT* carotid artery intima–media thickness

**Table 2:** *β* coefficient and 95% CI of serum 25(OH)D and arterial functional and structural properties

	*β* coefficient	95% CI	*p* value
Carotid artery distensibility	0.048	0.010, 0.096	0.04
Brachial artery distensibility	0.013	− 0.030, 0.056	0.135
Carotid artery IMT	− 0.002	− 0.003 to 0.000	0.048

The model is adjusted for age, sex, mean arterial pressure and heart rate

## Discussion

In the present study performed in older men and women referred to the outpatient clinic, serum 25(OH)D levels were associated with both carotid distensibility and carotid artery intima–media thickness. No association of vitamin D status with brachial distensibility was observed. The results from this cross-sectional study suggest that serum vitamin D levels could affect vascular structural and functional properties and that various vascular territories could be affected differently.

There is accumulating evidence that vitamin D deficiency is associated with an increased risk for cardiovascular disease. Large population based studies have previously shown an association between poor vitamin D status and an increased risk of cardiovascular events and cardiovascular related mortality, and have also suggested that vitamin D could be important in the prevention of vascular calcification [5]; a meta-analysis of 12 prospective studies underlined an increased 33% change for CHD in patients with the lowest quartile of vitamin D levels [6].

Arterial stiffness increases with age and is a predictor of morbidity and mortality [7]. Well established risk factors for arterial stiffening are hypertension, diabetes mellitus, inflammation [8], dyslipidemia [9].

However, a role for vitamin D in the process of arterial stiffening has also been suggested [10]; its deficiency has been associated with activation of the proinflammatory mechanism, promoting atherogenesis [11].

Several studies reported on the association between serum 25(OH)D levels and arterial stiffness. In the Baltimore Longitudinal Study of Aging, a prospective study of normative aging, vitamin D status was inversely associated with carotid–femoral pulse wave velocity (PWV) in a cohort of 1228 healthy volunteers with a mean age of 70 years [12]. A recent cross-sectional study by Mayer et al. in a relatively young population (mean age 52.8 years) reported an association between vitamin D status and aortic pulse wave velocity [13]. The exact role of vitamin D in cardiovascular health and specifically the role in arterial stiffening is thought to be multifactorial. Both animal and human studies have shown that vitamin D metabolites are a negative regulator of the renin–angiotensin–aldosterone system (RAAS) [14]. The RAAS is involved in maintenance of blood pressure, electrolyte homeostasis and control of intravascular volume. Large prospective studies have shown an association between low vitamin D levels and increased activity of RAAS which may result in hypertension and increased water intake and sodium absorption [14]. Furthermore, vitamin D metabolites are thought to directly alter myocyte contractility and proliferation [15]. Effects of vitamin D have also been reported on endothelial function, regulation of vascular endothelial growth factor (VEGF) production and on insulin and glucose handling [16]. It has previously been reported that vascular stiffening can differ between various vascular regions. In a study by Ruitenbeek et al. [17] age and blood pressure influenced functional properties of the elastic central arteries but not the muscular peripheral arteries. Based on the results of the present study, it can also be hypothesized that central and peripheral arteries are affected differently by vitamin D deficiency in regard to their functional and structural properties as associations are observed with carotid distensibility and IMT, while no association is observed with brachial distensibility.

The present study has some limitations. First, given the relatively small number of participants generalizability is limited. Second, the cross-sectional design limits conclusions on the causal relationship between vascular characteristics and vitamin D levels. Third, no information was available on the use of lipid lowers medication.

In conclusion, in a group of older men and women from the outpatient clinic, mean serum 25(OH)D levels were associated with both structural and functional properties of the carotid artery. We found no association between mean serum 25(OH)D levels and brachial artery distensibility.

## Abbreviations

VDR: Vitamin D receptor
PP: Pulse pressure
SBP: Systolic blood pressure
DBP: Diastolic blood pressure
MAP: Mean arterial pressure
*D*: End-diastolic diameter
Δ*D*: Absolute stroke change in diameter during systole
DC: Distensibility coefficient
CCA: Common carotid artery
c-IMT: Carotid artery intima–media thickness
PWV: Pulse wave velocity
anti-ht: Anti-hypertensive drugs
RAAS: Renin––angiotensin–aldosterone system
Ang II: Angiotensin II

## References

[1] Bikle D, Vitamin D (2014). Metabolism, mechanism of action, and clinical applications. Chem Biol.

[2] Reynolds JA, Haque S, Berry J, Pemberton P, Teh L-S, Ho P, Gorodkin R, Bruce IN (2012). 25-Hydroxyvitamin D deficiency is associated with increased aortic stiffness in patients with systemic lupus erythematosus. Rheumatology (Oxford).

[3] Oudshoorn C, van der Cammen TJ, McMurdo ME, van Leeuwen JP, Colin EM (2009). Ageing and vitamin D deficiency: effects on calcium homeostasis and considerations for vitamin D supplementation. Br J Nutr.

[4] Hoeks AP, Brands PJ, Smeets FA, Reneman RS (1990). Assessment of the distensibility of superficial arteries. Ultrasound Med Biol.

[5] Zittermann A, Schleithoff SS, Koerfer R (2007). Vitamin D and vascular calcification. Curr Opin Lipidol.

[6] Brondum-Jacobsen P, Nordestgaard BG, Schnohr P, Benn M (2013). 25-hydroxyvitamin D and symptomatic ischemic stroke: an original study and meta-analysis. Ann Neurol.

[7] Mattace-Raso FUS, van der Cammen TJ, Hofman A (2006). Arterial stiffness and risk of coronary heart disease and stroke: the Rotterdam Study. Circulation.

[8] Chen NX, Moe SM (2012). Vascular calcification: pathophysiology and risk factors. Curr Hypertens Rep.

[9] Norman PE, Powell JT (2014). Vitamin D and cardiovascular disease. Circ Res.

[10] Pradhan AD, Manson JE (2016). Update on the Vitamin D and OmegA-3 trial (VITAL). J Steroid Biochem Mol Biol.

[11] Nitsa A, Toutouza M, Machairas N (2018). Vitamin D in cardiovascular disease. In Vivo.

[12] Giallauria F, Milaneschi Y, Tanaka T (2012). Arterial stiffness and vitamin D levels: the Baltimore longitudinal study of aging. J Clin Endocrinol Metab.

[13] Mayer O, Filipovský J, Seidlerová J (2012). Tha association between low 25-hydroxyvitamin D and increased aortic stiffness. J Hum Hypertens.

[14] Tomaschitz A, Pilz S, Ritz E (2010). Independent association between 1,25-dihydroxyvitamin D, 25-hydroxyvitamin D and the renin-angiotensin system: the Ludwigshafen Risk and Cardiovascular Health (LURIC) study. Clin Chim Acta.

[15] Inoue T, Kawashima H (1988). 1,25-Dihydroxyvitamin D3 stimulates 45Ca2+-uptake by cultured vascular smooth muscle cells derived from rat aorta. Biochem Biophys Res Commun.

[16] Kim DJ (2017). Vitamin D and coronary atheroslerosis. Osteoporos Sarcopenia.

[17] Ruitenbeek AG, van der Cammen TJ, van der Meiracker AH, Mattace-Raso FU (2008). Age and blood pressure levels modify the functional properties of central but not peripheral arteries. Angiology.

